# Latent heterogeneity of deviant behaviors and associated factors among ethnic minority adolescents: a latent class analysis

**DOI:** 10.1186/s13034-024-00771-7

**Published:** 2024-07-31

**Authors:** Kunjie Cui, Ted C. T. Fong, Paul Siu Fai Yip

**Affiliations:** 1 Research Institute of Social Development, Southwestern University of Finance and Economics, Chengdu, Sichuan China; 2 Research Hub of Population Studies, The University of Hong Kong, Hong Kong, Hong Kong S.A.R.; 3 Centre on Behavioral Health, The University of Hong Kong, Hong Kong, Hong Kong S.A.R.; 4 Department of Social Work & Social Administration, The University of Hong Kong, Hong Kong, Hong Kong S.A.R.; 5 The HKJC Centre for Suicide Research and Prevention, The University of Hong Kong, 2/F, The HKJC Building for Interdisciplinary Research, 5 Sassoon Road, Pokfulam, Hong Kong, Hong Kong S.A.R.

**Keywords:** Deviant behavior, Ethnic minority adolescents, Latent class analysis, Mental health, Positive youth development, Risk factors, School belonging

## Abstract

**Background:**

Deviant behaviors are common during adolescence. Despite the diversity of juvenile delinquency, the patterns of deviant behaviors remain unclear in ethnic minorities. The present study aimed to evaluate the latent heterogeneity of deviant behaviors and associated factors in ethnic minority Yi adolescents.

**Methods:**

The present study recruited a large sample of 1931 ethnic minority Yi adolescents (53.4% females, mean age = 14.7 years, SD 1.10) in five secondary schools in 2022 in Sichuan, China. The participants completed measures on 13 deviant behaviors and demographic characteristics, attitudinal self-control, and psychological distress. Sample heterogeneity of deviant behaviors was analyzed via latent class analysis using class as the cluster variable.

**Results:**

The data supported three latent classes with measurement invariance by sex. 68.2%, 28.0%, and 3.8% of the sample were in the Normative, Borderline, and Deviant class, with minimal, occasional, and extensive deviant behaviors, respectively. The Deviant class was more prevalent in males (6.5%) than females (1.6%). There were significant class differences in domestic violence, school belonging, self-control, anxiety, and depressive symptoms. Males, domestic violence, low school belonging, and impaired self-control significantly predicted higher odds of the Deviant and Borderline classes compared to the normative class.

**Conclusion:**

This study provided the first results on three latent classes of deviant behaviors with distinct profiles in ethnic minority adolescents in rural China. These results have practical implications to formulate targeted interventions to improve the psycho-behavioral functioning of the at-risk adolescents in ethnic minorities.

**Supplementary Information:**

The online version contains supplementary material available at 10.1186/s13034-024-00771-7.

## Introduction

Adolescence is an important transitional life stage on the development of moral cognition and behaviors and adolescents are subject to various risk factors for delinquent behaviors [1]. Juvenile delinquency refers to adolescents’ engagement in deviant behaviors (such as fighting, cheating at school, and smoking) that violate social norms and values, and delinquent behaviors (such as theft, illicit drug use, and vandalism) that are illegal [2]. Juvenile delinquency is a growing societal issue around the world including China [3, 4], with potential impacts in terms of economic costs, public safety, and social dislocation. In the Chinese context, the minimum legal age for purchasing tobacco products is 18 years old. Smoking is commonly known as a harmful and addictive habit with various adverse health effects.

Deviant behaviors in adolescents have been associated with factors in the individual, family and school domains [5]. Empirical studies have found that lack of self-control is a key determinant of deviant behaviors in Chinese adolescents [6–8]. Direct and vicarious violent victimization, including experiences of domestic violence and witnessing violence, has been linked to an increased risk of juvenile delinquency [9]. Family functioning have been negatively correlated with deviant behavior [10] and family conflicts have been positively associated with mental health symptoms among Chinese adolescents [11].

School life plays an essential role in the academic, personal, and social development of adolescents [12]. A longitudinal study has linked lower levels of school belonging with higher rates of juvenile delinquency in American youths [13]. Besides, cultural factors, such as being part of an ethnic minority, could play a contributory role in deviant behaviors among adolescents. Ethnic minority status refers to being part of an ethnic group that is characterized by distinct cultural or ethnic attributes within the Chinese population. The relationship between deviant behaviors in adolescence and ethnic minority status is a complex and multifaceted issue. Research has shown higher rates of deviant behaviors among ethnic minority groups [14]. The relationships between deviant behaviors and ethnic minority status could be attributed to various factors in the economic (socioeconomic disadvantages), social (perceived prejudice and discrimination), cultural (cultural dissonance) domains [15–18].

The Liangshan Yi Autonomous Prefecture in China comprises the largest population of ethnic Yi in China. Ethnic minorities in this area faced various social problems such as severe poverty, limited access to resources and opportunities, and rampant crime over the past century [19, 20]. In this region, parents tend to have lower education levels and often have to work outside the home for extended periods of time. These can make it challenging for them to closely monitor their children’s behavior and might contribute to lower levels of social control over adolescents. Ethnic minority adolescents in China have shown higher rates of illicit drug use and property delinquency than Han adolescents [21, 22]. A recent study conducted among Yi families in Liangshan highlights the need to provide culturally sensitive support among ethnic minority adolescents during family ethnic socialization [23]. Given the isolated geographical locations and mountainous terrain of this prefecture, it is a common practice of sending ethnic minority students away from their homes to attend boarding schools. This highlights the importance of school context for behavioral, cognitive, and psychological development of adolescents [24, 25]. A recent study has linked ethnic identity with mental health symptoms in ethnic minority Yi students [26]. Despite the potential importance and diversity of cultural contexts across ethnic minority groups, few studies in the Chinese context have investigated the heterogeneity of deviant behaviors in the context of ethnic minority adolescents.

Deviant behaviors in adolescents are often complex and multifaceted. Latent class analysis (LCA) is a person-oriented modeling technique that can identify distinct subgroups of adolescents based on their patterns of deviant behaviors [27]. This analytical approach is useful for examining the heterogeneity in deviant behaviors and informing the associated risk factors. A literature search on Web of Science on 7th January 2024 using keywords of (“juvenile delinquency” or “deviant behaviour” or “delinquent behaviour” or “antisocial behavior” or “risk behavior” (Title) and latent class (Title)) found 15 articles that used LCA to examine the latent heterogeneity of deviant behavior in samples of adolescents and sexual minority youths. None of these studies, however, are based on ethnic minority adolescents. In the Chinese context, recent studies [28, 29] have yet to utilize LCA to examine the latent heterogeneity of deviant behaviors of adolescents, particularly among ethnic minority adolescents.

In light of the research gaps, the present study first aimed to investigate the latent heterogeneity of deviant behaviors in ethnic minority adolescents in China. The second objective was to examine the differences among the derived latent classes in associated variables, including demographic, family, school characteristics, self-control, and mental health symptoms. This would lead to a better understanding of the risk factors of deviant behaviors among Chinese ethnic minority adolescents. There are two hypotheses in the present study. First, considering existing LCA findings in mainstream adolescents [30–33], we hypothesized that there would exist multiple latent classes of deviant behaviors in ethnic minority adolescents. Second, the derived latent classes would demonstrate significant profile differences in associated variables, as found in previous similar studies among children and runaway youths [34, 35].

## Methods

### Participants

In the present study, 1931 ethnic Yi adolescents aged 12 to 17 were recruited in the Liangshan Prefecture of Sichuan, China from June to July 2022. The overall response rate of the adolescents was 86.8%. The participants were recruited from 38 classes in five secondary schools with an average class size of 51 students. Inclusion criteria were ethnic minority status, Grade 7 or 8, and able to understand simplified Chinese. The study purpose was clearly explained to the adolescents and written informed consent was obtained from the adolescents and their legal guardians. Study participation was voluntary and the adolescents could withdraw from the study at any time without negative consequence. The collected information was anonymized to preserve data confidentiality. Ethical approval was obtained from the Human Research Ethics Committee of the first author’s university.

The required sample size was calculated according to Dziak, Lanza and Tan [36] where N = m^(w2)^_80_/w^2^. In this equation, the numerator denoted the value of N × w^2^ for which power exceeded 80% and the denominator denoted the square of effect size. For 13 binary items, m^(w2)^_80_ was estimated to be 62.3 and 57.1 for 3 and 5 classes, respectively. For w = 0.2 (low to medium values), the required sample size was 1558. The present sample size (N = 1931) provided at least 80% power for13-item, 3-class to 5-class LCA with w = 0.2.

### Measures

The study questionnaire included questions on deviant behaviors, demographic, family and school characteristics, attitudinal self-control, and mental health. It was field tested in May 2022 and underwent minor revisions based on feedback from the adolescents.

In this study, deviant behaviors referred to engagement in behaviors that causes harm to others or property or puts oneself in a position that invites harm from being compromised/inebriated, or break certain rules, regulations or norms. *Deviant behaviors* were assessed via 13 binary (yes/no) items on the respondents’ engagement in deviant behaviors over the past year: stealing others’ properties (thief), vandalism of properties (vandalism), robbery of others’ properties (robbery), fighting, obtain money or property by fraud (fraudulence), playing online games for more than 5 h continuously (gaming addiction), cheating, runaway, truancy, drinking alcohol, illicit drug use, smoking, and gambling. The list of deviant behaviors was developed based on existing literature on juvenile delinquency in Chinese adolescents [11, 37, 38] and is shown in Supplementary Table 1. We adapted and modified the items based on the Code of Conduct for Middle School Students and Law on the Prevention of Juvenile Delinquency in China to fit the Chinese ethnic contexts. The 1-factor model on deviant behaviors showed adequate model fit to the present sample with comparative fit index (CFI) = 0.98, root-mean-square error of approximation (RMSEA) = 0.036, and standardized root mean square residual (SRMR) = 0.050. The 13 items had strong factor loadings (λ = 0.61–0.95, *p* < 0.01) and good reliability (Cronbach’s α = 0.89) in the present sample.

*Demographic characteristics* assessed in the questionnaire included biological sex, age, religious belief, and urban registration (versus rural). *Family characteristics* included having a single parent and domestic violence via three self-constructed items such as “Family members are loudly reprimanded, insulted or humiliated” on a 5-point Likert scale from 1 = “never” to 5 = “always”. *School belonging* was assessed by six self-constructed items such as “I think I belong to the school I am currently at” and “My school is a great place” on a 5-point Likert scale from 1 = “strongly disagree” to 5 = “strongly agree”. The 1-factor model on school belonging showed satisfactory model fit to the present sample with comparative fit index (CFI) = 0.98, root-mean-square error of approximation (RMSEA) = 0.068, and standardized root mean square residual (SRMR) = 0.028. Acceptable to good reliability was found for the total score of domestic violence (α = 0.67) and school belonging (α = 0.86).

*Attitudinal self-control* was assessed via the Grasmick Low Self-Control Scale [39], which is a 23-item scale on six types of self-control trait. The present study included four subscales: impulsivity (3 items), risk seeking (4 items), self-centeredness (4 items), and temperament (4 items). The items are scored on a 4-point scale from 1 = “strongly disagree” to 4 = “strongly agree”. The Low Self-Control scale has been validated among adolescent samples in the Chinese context with good psychometric properties [8]. The 4-factor model showed good model fit to the data with CFI = 0.97, RMSEA = 0.036, and SRMR = 0.030 in the present sample with acceptable reliability (α = 0.64–0.81).

*Anxiety* and *depressive symptoms* were measured by the Generalized Anxiety Disorder-7 (GAD-7) and Patient Health Questionnaire-9 (PHQ-9), respectively, over the past two weeks [40, 41]. The items are rated on a 4-point Likert scale from 0 = “not at all” to 3 = “nearly every day”. The composite score for GAD-7 and PHQ-9 ranged from 0 to 21 and from 0 to 27, respectively. The cutoff scores for mild, moderate, and moderately severe anxiety/depression were represented by GAD-7/PHQ-9 scores of 5, 10, and 15, respectively. Both the GAD-7 and PHQ-9 have been validated in the Chinese context [42, 43]. The 1-factor model showed good model fit to the present sample for both GAD-7 with CFI = 0.99, RMSEA = 0.027, and SRMR = 0.013 and PHQ-9 with CFI = 0.95, RMSEA = 0.059, and SRMR = 0.034 with good reliability (α = 0.88–0.91).

### Data analysis

Prevalence of deviant behaviors was compared across sex via chi-square tests. There were no missing data in the 13 deviant behaviour items. The 13 deviant behaviors showed a mean intraclass correlation of 0.077 within the class. Given the clustered nature, LCA was conducted under the TYPE = COMPLEX option with class as a cluster variable in Mplus 8.6 to adjust for non-independence in chi-square statistic and standard errors. The LCA model assumes that the observed variables are categorical, and that the latent classes are mutually exclusive and exhaustive. The deviant behaviors were assumed conditionally independent of each other within each latent class [44].

One-class to 5-class LCA models were estimated on the 13 deviant behaviors using robust maximum likelihood estimator [45]. Model fit was evaluated using Bayesian information criterion (BIC), sample-size adjusted Bayesian information criterion (aBIC), and bivariate log-likelihood chi-square (TECH10) with lower values indicating better fit. The Lo–Mendell–Rubin (LMR) likelihood ratio test [46] compared the fit of the k-class LCA model to the alternative k-1 class model with a *p*-value < 0.05 favoring the k-class model. Model classification quality was assessed using entropy and average latent class probabilities, with values of at least 0.80 and 0.90 indicating adequate classification, respectively. Sample heterogeneity in deviant behaviors was reported via prevalence and conditional item probabilities of latent classes. Statistical significance was set at *p* < 0.01 level in this study to address multiple testing.

LCA models were estimated separately by sex to test the stability of latent class structure. Measurement invariance was evaluated by comparing BIC of the LCA models with and without equality constraints on the item thresholds. The latent classes were compared in terms of demographic, family, and school characteristics, attitudinal self-control, and mental health symptoms. Class differences were estimated using the Bolck, Croon, and Hagenaars (BCH) method [47] with post-hoc comparisons using Sidak correction. 3-step multinomial logistic regression was conducted on the latent class memberships using demographic, family, school characteristics, and low self-control as predictors. The associations were estimated by adjusted odds ratios (OR), which were regarded as statistically significant if the 95% confidence interval (CI) excluded one.

## Results

### Sample characteristics

As Table 1 shows, half (53.4%) of the sample was females, and the average age was 14.7 years (SD 1.08). Most of them had a rural registration (95.7%) and one-eighth (13.1–13.2%) of them had religious belief and a single parent. Overall, the levels of school belonging, and self-control of the sample were moderate. The mean score for GAD-7 and PHQ-9 was 4.77 (SD 4.51) and 6.61 (5.08), respectively. The proportion of adolescents with minimal, mild, moderate, and moderately severe anxiety symptoms was 53.8%, 33.5%, 9.0%, and 3.7%, respectively. The corresponding proportion was 37.6%, 41.3%, 13.9%, and 7.1%, respectively, for depressive symptoms. As Supplementary Table 1 shows, the prevalence of deviant behaviors ranged from 2.8 to 31.6%. Higher prevalence (29.6–31.6%) was found for cheating at school and gaming addiction; while illicit drug use, robbery, fraudulence, and gambling were among the least common (2.8–5.3%). Males showed significantly higher prevalence in most deviant behaviors (χ^2^(1) = 15.2–258.0, *p* < 0.01) except for cheating and runaway than females.

**Table 1:** Demographic profiles and descriptive statistics of the sample (N = 1931)

Categorical items	Yes (%)
Male	46.6
Have religious belief	13.1
Urban registration	4.3
Single parent	13.2

Continuous items	Range	Mean (SD)
Age (years)	12–17	14.7 (1.08)
Domestic violence	1–5	1.27 (0.50)
School belonging	1–5	3.66 (0.84)
Impulsivity	1–4	2.06 (0.68)
Risk seeking	1–4	1.77 (0.66)
Self-centeredness	1–4	1.63 (0.65)
Temperament	1–4	1.91 (0.66)
Anxiety symptoms	0–21	4.77 (4.51)
Depressive symptoms	0–27	6.61 (5.08)

Higher scores indicating better school attachment, more severe domestic violence, and higher levels of impulsiveness, risk-seeking, self-centeredness, temperament, and anxiety and depressive symptoms

### Latent class analysis models

Table 2 shows the fit indices of the LCA models in the whole sample and across sex. Substantial decreases in BIC and aBIC were found from 1-class to 3-class LCA models, after which the ICs levelled off. The 3-class, 4-class, and 5-class LCA models showed comparable BIC and the 3-class LCA model showed the minimum BIC in the male and female subsamples. The 4-class and 5-class LCA models showed lower bivariate log-likelihood chi-squares with fewer significant standardized residuals. The LMR likelihood ratio test results consistently pointed to the 3-class solution in the whole sample and across sex with a significantly better fit than the 2-class solution and comparable model fit as the 4-class solution (*p* = 0.33–0.59). Moreover, the 3-class LCA model showed higher entropy (0.83–0.84) than the 4-class model (0.72–0.80). These findings supported the 3-class LCA model in terms of BIC, LMR test, and entropy. The multiple-group LCA model with sex-invariant item thresholds showed comparable model fit in terms of BIC (18,069 versus 18,064) as the configural model with non-invariant item thresholds, supporting measurement invariance of latent class structure across sex.

**Table 2:** Fit indices of latent class models on deviant behaviors in the sample and across sex

Model	#	BIC	aBIC	LMR	Entropy	Bivariate LL χ^2^ (N)
Whole sample						
1-class	13	18,796	18,754	–	–	9991 (284)
2-class	27	16,042	15,956	< 0.01	0.88	890.4 (67)
3-class	41	15,534	15,404	< 0.01	0.84	140.4 (6)
4-class	55	15,527	15,353	0.33	0.72	106.0 (3)
5-class	69	15,537	15,318	0.50	0.79	55.8 (0)
Male						
1-class	13	10,451	10,410	–	–	5433 (276)
2-class	27	8986	8900	< 0.01	0.87	497.7 (39)
3-class	41	8727	8597	< 0.01	0.83	101.3 (6)
4-class	55	8747	8572	0.43	0.80	76.4 (4)
5-class	69	8770	8551	0.65	0.80	42.6 (1)
Female						
1-class	13	7689	7648	–	–	3561 (190)
2-class	27	6771	6685	< 0.01	0.87	432.6 (31)
3-class	41	6604	6474	0.024	0.84	64.1 (2)
4-class	55	6654	6480	0.59	0.74	52.7 (0)
5-class	69	6711	6492	0.76	0.74	33.8 (0)

Lower values of BIC, aBIC, bivariate LL, N, and LMR (*p* < 0.05) indicate better model fit and higher values of entropy indicate better classification

^#^ Number of free parameters, *BIC* Bayesian information criterion, *aBIC* sample-size adjusted BIC, *LMR* Lo-Mendell–Rubin likelihood ratio test, *LL* log likelihood, *N* overall number of significant standardized residuals

**Fig. 1:**
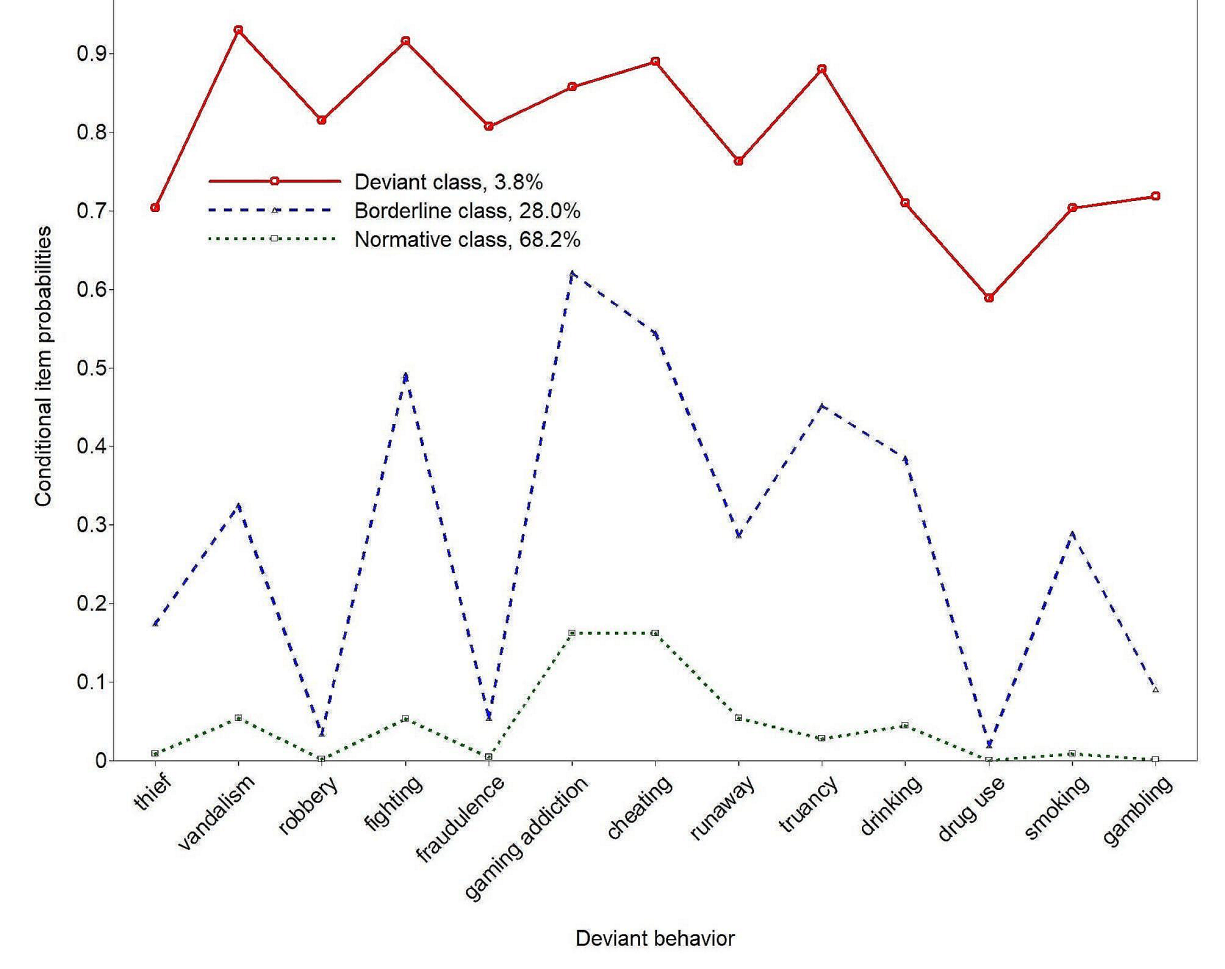
Conditional item probabilities of the 13 deviant behaviors for the 3-class latent class model

As Fig. 1 shows, the normative class comprised 68.2% of adolescents and had very low probabilities (0.0–5.4%) in all deviant behaviors except for gaming addiction and cheating (16.2%). The Borderline class comprised 28.0% of adolescents and showed substantial probabilities in fighting, gaming addiction, cheating, and truancy (45.2–62.0%). This class showed non-negligible probabilities in vandalism, runaway, drinking, and smoking (28.6–38.5%) but low probabilities in thief, robbery, fraudulence, illicit drug use, and gambling (1.9–17.4%). The smallest class comprised 3.8% of adolescents and was labelled the Deviant class. This class had the highest conditional item probabilities (58.9–93.0%) of all deviant behaviors.

### Comparison of latent classes

As Table 3 shows, males were significantly more prevalent (*p* < 0.01) in the Deviant and Borderline class than normative class. No significant differences (χ^2^(2) = 1.32–2.70, *p* = 0.26–0.52) were found in other demographic variables. Significant differences (χ^2^(2) = 42.6–147.9, *p* < 0.01) were found across the three classes in domestic violence, school belonging, self-control, GAD7, and PHQ9 symptoms. Both Borderline and Deviant classes showed significantly lower levels of school belonging and self-control and higher levels of domestic violence, and GAD7 and PHQ9 symptoms than the Normative class. Moreover, the Deviant class showed significantly lower levels of school belonging and higher levels of domestic violence, risk seeking, self-centeredness, and GAD7 and PHQ9 symptoms than the Borderline class. Supplementary Table 2 shows significant differences (*p* < 0.01) in the prevalence of at least moderate anxiety symptoms (Normative class: 7.7%; Borderline class: 18.9%; Deviant class: 51.5%) and moderate depressive symptoms (Normative class: 12.9%; Borderline class: 32.8%; Deviant class: 67.9%).

**Table 3:** Comparison of demographic, family, and school characteristics, attitudinal self-control, and mental health symptoms across the three latent classes of deviant behaviors

Variables	Normative class (N = 1317, 68.2%)	Borderline class (N = 541, 28.0%)	Deviant class (N = 73, 3.8%)	Overall 3-class difference	
	Mean/% (SE)	Mean/% (SE)	Mean/% (SE)	χ^2^	p
Male	36.5% (2.0)^a^	66.9% (2.7)^b^	79.3% (4.8)^b^	136.7	< 0.01
Have religious belief	13.6% (2.3)	12.6% (2.1)	7.4% (3.7)	2.70	0.26
Urban registration	3.9% (0.9)	4.9% (1.3)	7.6% (3.8)	1.40	0.50
Single parent	12.4% (1.5)	14.6% (2.5)	17.8% (5.7)	1.32	0.52
Age, years	14.6 (0.08)^a^	14.9 (0.08)^b^	14.7 (0.10)^ab^	9.73	< 0.01
Domestic violence	1.17 (0.02)^a^	1.38 (0.03)^b^	2.14 (0.12)^c^	86.8	< 0.01
School belonging	3.83 (0.06)^c^	3.33 (0.07)^b^	3.03 (0.11)^a^	110.5	< 0.01
Impulsivity	1.97 (0.03)^a^	2.23 (0.04)^b^	2.31 (0.09)^b^	42.6	< 0.01
Risk seeking	1.65 (0.03)^a^	1.97 (0.03)^b^	2.33 (0.09)^c^	132.2	< 0.01
Self-centered	1.52 (0.03)^a^	1.80 (0.04)^b^	2.33 (0.09)^c^	76.4	< 0.01
Temper	1.78 (0.03)^a^	2.14 (0.03)^b^	2.33 (0.08)^b^	110.8	< 0.01
Anxiety symptoms	3.79 (0.19)^a^	6.34 (0.20)^b^	9.47 (0.58)^c^	147.9	< 0.01
Depressive symptoms	5.52 (0.21)^a^	8.63 (0.33)^b^	10.9 (0.76)^c^	86.0	< 0.01

*SE* standard error

^abc^Significant post-hoc differences among the 3 latent classes are indicated by their superscripts with a < b < c; Equality of means and proportions across classes were tested by Bolck, Croon, and Hagenaars (BCH) procedure. The range of the continuous variables are shown in Table 1

### Predictors of latent classes memberships

In Table 4, having a single parent was not significantly associated (*p* > 0.05) with the latent class memberships. Males had significantly higher odds in Borderline class (OR = 4.80, 95% CI 3.46–6.64, *p* < 0.001) and Deviant class (OR = 10.6, 95% CI 4.32–26.3, *p* < 0.001) compared to Normative class than females. Compared to Normative class, school belonging was significantly associated with lower odds of Borderline class (OR = 0.47, 95% CI 0.37–0.60, *p* < 0.001) and Deviant class (OR = 0.42, 95% CI 0.30–0.59, *p* < 0.001). Temperament were significantly associated with higher odds of Borderline class (OR = 1.97, 95% CI 1.44–2.68, *p* < 0.001) and Deviant class (OR = 1.92, 95% CI 1.20–3.07, *p* < 0.01) compared to Normative class. Domestic violence was significantly associated with higher odds of Deviant class compared to Normative class (OR = 6.52, 95% CI 3.54–12.0, *p* < 0.001) and Borderline class (OR = 2.57, 95% CI 1.78–3.72, *p* < 0.001). Risk-seeking was significantly associated with higher odds of Borderline class (OR = 1.34, 95% CI 1.10–1.63, *p* < 0.01) compared to Normative class. Self-centeredness was significantly associated with higher odds of Deviant class (OR = 2.25, 95% CI 1.32–3.84, *p* < 0.01) compared to Borderline class. Sensitivity analysis was conducted by including religious belief and urban registration as control variables in the multinomial logistic regression and the results were highly similar to those in Table 4.

**Table 4:** 3-step multinomial logistic regressions of latent class memberships of deviant behaviors on demographic, family, and school characteristics, and attitudinal self-control

	Borderline class	Deviant class	Deviant class
Reference class	Normative class	Normative class	Borderline class
Factors	OR (95% CI)	OR (95% CI)	OR (95% CI)
Male	4.80*** (3.46–6.64)	10.6*** (4.32–26.3)	2.22 (0.96–5.16)
Age, years	1.26* (1.05–1.51)	1.15 (0.89–1.47)	0.91 (0.72–1.16)
Single parent	1.26 (0.73–2.17)	2.24 (0.86–5.83)	1.79 (0.70–4.58)
Domestic violence	2.53*** (1.50–4.27)	6.52*** (3.54–12.0)	2.57*** (1.78–3.72)
School belonging	0.47*** (0.37–0.60)	0.42*** (0.30–0.59)	0.88 (0.67–1.17)
Impulsivity	1.41 (1.07–1.86)	0.90 (0.44–1.84)	0.64 (0.32–1.30)
Risk seeking	1.34** (1.10–1.63)	1.70 (1.01–2.86)	1.27 (0.73–2.20)
Self-centeredness	0.85 (0.62–1.17)	1.92 (1.16–3.18)	2.25** (1.32–3.84)
Temperament	1.97*** (1.44–2.68)	1.92** (1.20–3.07)	0.98 (0.64–1.49)

**p < 0.01; ***p < 0.001; *OR* odds ratios; for continuous independent variables, ORs are per score; CI confidence interval; Male and single parent were categorical variables and the other seven variables were continuous variables

## Discussion

The present study characterized the latent heterogeneity of deviant behaviors and reported the first results on the three latent (Normative, Borderline, and Deviant) classes in a minority population of Chinese adolescents. The three latent classes showed distinct profiles of deviant behaviors and significant relationships with external variables. The majority of the adolescents were in the Normative class with minimal probabilities of deviant behaviors, higher levels of school belonging and self-control and lower levels of domestic violence and mental distress. A quarter of the sample belonged to the Borderline class with moderate probabilities in deviant behaviors (i.e. fighting, gaming addiction, cheating, and truancy). A longitudinal study has found temporal associations between deviant behaviors and school engagement and community violence exposure [48]. Further longitudinal research is needed to track the developmental trajectories of deviant behaviors among the adolescents and investigate potential risk factors of the transition from the Borderline class to the Deviant class in the future.

The Deviant class showed high probabilities in all deviant behaviors. Despite its low prevalence (3.8%), adolescents in this class showed the highest exposure to domestic violence and impairment in school belonging and self-control. More than half of this class had at least moderate levels of anxiety and depressive symptoms, which indicated substantial mental health needs for this class with poorer family and school functioning. The present study found a higher prevalence of Deviant class in males than females. This is consistent with the sex difference in deviant behaviors found in adolescents with higher levels of sensation-seeking and lower levels of impulse control in young men than young women [49, 50]. Existing studies [51, 52] have shown potential impact of domestic violence on self-control among children and adolescents. On the one hand, exposure to violence could decrease self-regulation abilities. On the other hand, low self-control could aggravate conflict within the family and contribute to domestic violence. Future research should elucidate the possible bidirectional relationships between domestic violence and self-control.

The present study identified factors associated with the latent class of deviant behavior. Our positive associations between impaired self-control and deviant behavior are in line with previous findings [53]. While risk-seeking and temperament were found to distinguish the Normative class from the other two classes, adolescents who were more self-centeredness showed significantly higher odds in Deviant class relative to Borderline class. Thus, interventions aimed at promoting self-control and reducing self-centeredness could be implemented both within families and schools. Family cohesion programs could be implemented to promote positive family relationships and reduce the risk of domestic violence. Positive youth development programs could be implemented in schools to promote self-control and a sense of school belonging among adolescents [54, 55]. These interventions could potentially help reduce the risk of deviant behavior among ethnic minority adolescents.

Throughout history, Chinese ethnic minorities have exhibited unique behavioral characteristics. Few studies have systematically analyzed their patterns of deviant behaviors and associated risk factors in social and individual domains. As Yi [56] pointed out, arbitrarily attributing poor minority performance to a striking feature of mainstream discourse is incorrect. This can lead to attributing the poverty and delinquency of the ethnic minority individuals solely to their cultural and social characteristics, which can create more vicious circles based on their psychosocial symptoms. In comparison to assimilation, the ethnic minority in China has been delineated as experiencing “borderline integration” [57]. To avoid intensifying the marginalization of ethnic minorities within the wider society, systematic research is necessary to elucidate the psycho-behavioral characteristics of ethnic minorities, identify potential risk and protective factors, and inspire more feasible intervention practices for their development in China. Further studies should compare the deviant behaviors of adolescents across ethnicity groups and investigate the role of acculturation in the well-being of adolescents across diverse ethnic backgrounds [58–60].

In the past decade, various poverty alleviation and rural revitalization policies have been implemented in the ethnic minority areas in China, which led to rapid development and substantial improvements in material lives [61, 62]. Apart from economic development of impoverished regions, it is crucial to enhance psycho-behavioral well-being of the adolescents for a holistic development. Our study provides insights into interventions for deviant behaviors among ethnic minority adolescents. Policymakers and practitioners should prioritize adolescents with lower levels of school belonging, exposure to domestic violence, higher levels of mental distress and tendencies towards risk-seeking and self-centeredness, and temperament. Our findings call for a greater emphasis to promote mental wellness among ethnic minorities. This could be achieved via adjustment of cultural and societal norms and beliefs about family structures, roles, and dynamics within ethnic minority communities to foster inclusive and harmonious family environments. A supportive school environment should be constructed by introducing professional teaching staff, educational psychologists, and school social workers in ethnic minority contexts. A comprehensive education curriculum should not only focus on academic achievement but also on the personal and psycho-behavioral development of adolescents to enhance their overall school learning experience.

## Limitations and future directions

There were several limitations in this study. First, the cross-sectional design did not permit inference of the directional relationship between deviant behaviors and school belonging and mental distress. Deviant behaviors could lead to less school belonging and greater mental health symptoms. Longitudinal studies are needed to clarify the temporal associations between deviant behaviors with associated factors and development of deviant behaviors across adolescence and early adulthood. Second, similar to other Chinese studies [63, 64], the present study assessed deviant behaviors using a self-constructed scale. Despite the adequate psychometric properties, our new instrument was examined only based on an ethnic minority sample, so its usefulness as an assessment tool of deviant behaviors was restricted to this population and our present findings may not generalize to other populations. Measurement invariance and normative scores should be established across ethnicity groups before valid comparisons could be made with data from other populations. Third, all the study variables were self-reported by the adolescents, which may introduce common method bias. The non-random sampling of the present ethnic Yi sample implies potential selection and response biases. The results might not generalize to other ethnic minority groups. Future studies should incorporate assessments rated by parents and teachers and objective biomarkers to improve the validity of the measures.

Fourth, the LCA model assumed the observed variables to be conditionally independent within each latent class. However, the relationships among deviant behaviors might not be solely explained by the latent class variable. Violation of this assumption could bias the latent class structure, class assignment, and parameter estimates. Future studies could estimate the significant standardized residuals in the LCA model as sensitivity analyses to replicate the present results. Fifth, though our study analysis properly accounted for the clustering nature of adolescents nested within the class, the present sample was recruited from only five schools. The low school size did not permit us to systematically examine the variation in deviant behavior at the school level. Further large-scale studies are recommended among adolescents from more schools to conduct multilevel LCA in deviant behaviors at the school level. Sixth, adolescents could engage in deviant behaviors due to peer influence and social pressure. The present study did not examine peer influence as a motive for deviant behaviors. Future studies should examine the associations between social factors (peer influence and bullying victimization) and macro-level factors (stereotypes and culture) and deviant behaviors in the Borderline and Deviant classes.

### Supplementary Information

Supplementary material 1

Supplementary material 2

## Data Availability

All data analysed during this study are included in this published article as supplementary materials. The Mplus syntax scripts are available from the first authors upon request by email.
