# Supplementary material for: Latent heterogeneity of deviant behaviors and associated factors among ethnic minority adolescents: a latent class analysis
Source: Child Adolesc Psychiatry Ment Health. 2024 Jul 31;18:93. doi: 10.1186/s13034-024-00771-7 (PMC11293068; doi:10.1186/s13034-024-00771-7)
Supplement: Supplementary file 1 — Supplementary material 1 [file 13034_2024_771_MOESM1_ESM.pdf]

Supplementary Table 1. Prevalence of deviant behaviors in male and female participants

Delinquent behaviors (yes %):	Whole sample	Female	Male	χ^2
1. Thief	8.1%	5.3%	11.3%	23.2***
2. Vandalism	16.3%	12.6%	20.5%	21.6***
3. Robbery	4.1%	2.4%	6.0%	15.7***
4. Fighting	20.8%	10.0%	33.2%	156.3***
5. Fraudulence	4.9%	2.3%	7.8%	30.8***
6. Gaming addiction	31.6%	15.7%	49.8%	258.0***
7. Cheating at school	29.6%	27.7%	31.8%	3.87
8. Runaway	14.6%	13.1%	16.2%	3.68
9. Truancy	17.8%	12.5%	23.9%	42.4***
10. Drinking	16.4%	13.3%	19.9%	15.2***
11. Drug use	2.8%	1.2%	4.6%	20.7***
12. Smoking	11.3%	5.1%	18.3%	83.3***
13. Gambling	5.3%	2.1%	8.9%	44.1***

*** $p < 0.001$; ** $p < 0.01$; N = 1931/900/1031 for whole sample/males/females. The items are presented in the order of the questionnaire.

Supplementary Table 2. Comparison of severity of anxiety and depressive symptoms across the three latent classes of deviant behaviors

Anxiety symptoms:	Normative class (68.2%)	Borderline class (28.0%)	Deviant class (3.8%)	χ^2
Minimal (0 – 4)	63.1%	38.0%	12.2%	195.7***
Mild (5 – 9)	29.2%	43.1%	36.3%	
Moderate (10 – 14)	5.2%	13.3%	41.1%	
Moderately severe/severe (15 – 21)	2.5%	5.6%	10.4%	
Depressive symptoms:	Normative class	Borderline class	Deviant class	χ^2
Minimal (0 – 4)	47.1%	20.1%	12.0%	256.8***
Mild (5 – 9)	39.9%	47.1%	20.1%	
Moderate (10 – 14)	8.5%	21.2%	49.2%	
Moderately severe/severe (15 – 27)	4.4%	11.6%	18.7%	

*** $p < 0.001$; N = 1849 for anxiety and depressive symptoms.
